# 

**Published:** 1911-10-15

**Authors:** 


### ANNOUNCEMENTS.

New york seventh and eighth district societies. The Union meeting of the Seventh and Eighth District Dental Societies will be held this year in the Hotel Statler, Buffalo, N. Y., November 9, 10, 11.

The meeting will close Saturday evening, November 11, with an open Oral Hygiene meeting, held in Convention Hall.

Already essayists and speakers of national reputation have been obtained and everything promises for a big successful meeting.

Be sure to be with us.

A. F. Isham,
J. P. Mallory,
M. A. Gregg,
J. T. McIntee,
C. A. Bennett,
M. C. Bradley,
Business Committee.

Michigan Board of Examiners. The next regular meeting of the Michigan State Board of Dental Examiners will be held at the Dental College, Ann Arbor, commencing Monday, November 13th, at 8:00 A. M., and continuing through the 18th. For application blanks, and full particulars address,

A. W. Haidle, *Sec.*,
Negaunee, Mich.

National Dental Association.—meeting of the executive council. There will be a meeting of the Executive

Council of the National Dental Association November 4th, 10:00 A. M., at the New Willard Hotel, Washington, D. C.

This meeting is called for the purpose for selecting Section Officers and Committees for the coming year, and was authorized by the Association at the Cleveland meeting. Members desiring to offer suggestions or recommendations in this connection, should promptly communicate same to any member of the Council, or the undersigned.

H. C. Brown, *Rec. Sec'y,*
185 East State St.,
Columbus, Ohio.

---

National Dental Association.—notice to the profession. In justice to the National Dental Association and Dr. C. N. Johnson, and the profession generally, it has been deemed advisable to send a statement to all the Dental Journals for publication in their October issues, in order that the profession may be correctly informed concerning a widely circulated report that the National Dental Association took official action at the Cleveland meeting, condemning tooth powders, pastes and mouth washes.

Dr. C. N. Johnson, of Chicago, read a paper at the meeting entitled "Mistakes, Common and Uncommon," from which the following is quoted, this being the only part of his paper which refers in any way to the misinterpreted quotation: "And in this connection there is another mistake which the profession is fostering and to which attention should be called. This is the constant use of mouth washes among our patients irrespective of whether or not they are indicated. I am far from condemning the legitimate use of carefully prepared stimulating and antiseptic solutions in those cases where the abnormal conditions in the mouth indicate such treatment, but mouth washes should be limited to their proper indications and should be eliminated from use in healthy mouths. Their use in these cases has a tendency to do more harm than good. If the mouth is healthy there is always the danger of disturbing the normal balance by the introduction of agents which tend to interfere with function. The maintenance of function is the surest way to health, and consequently the healthiest mouth is the one which function is most fully performed. The best stimulant for the gum tissue and the best polisher of enamel is the friction of food, in the function of mastication, but unfortunately mastication is not always carried out to its fullest efficiency. Therefore we use tooth brushes to make up this dereliction, and the moment we begin to depend upon solutions and washes to do the work which should be done mechanically by mastication and the brush that moment the tissues begin to deteriorate. I am not arguing against the use of a wash where the tissues are inflamed and the gums puffed and swollen, nor am I criticizing, as some have done, the mouth washes which are on the market to-day, many of which seem to be carefully compounded, but I venture to suggest that if the patient could be prevailed upon to have all irritants removed from the teeth in the way of deposit and the gums properly stimulated by massage and the friction of food and the brush that the restoration to health would be just as certain as by the use of drugs and it would be more permanent. But just here is the rub. It seems so hard to get the average individual to properly care for the teeth and gums by mechanical means unless some powder, paste or wash is prescribed as an incentive. In this particular these agents have been useful. They have been the means of inducing many negligent individuals to take care of the mouth, when otherwise it would have gone neglected. Most of them are prepared with a pleasant flavor and they leave the mouth with a refreshed and wholesome feeling as is so often expressed by the patients.

But the whole tendency of our teaching should be in the direction of preserving the normal balance between function and health rather than glossing over conditions by a false reliance on drugs. It is too much akin to the habit of using powerful and not always pleasant perfumes as a substitute for good honest cleanliness of person. The individual who carries with him the odor of the bath has little need for musk, and he whose teeth and mouth are kept constantly clean is seldom obliged to resort to the "dopeing" of drugs."

In addition to this the official stenographer writes that one of the discussers stated "that water was the best thing to use in brushing the teeth."

This paper was read before Section two and did not come before the Association in any other way, and no official action, as some of those reports would seem to convey was taken by the Association.

Fraternally yours,

A. R. Melendy, *President.*

H. C. Brown, *Rec. Sec'y.*

We publish the above at the request of Secretary Brown; but for the life of us, we can not see how any one that can interpret the English language can distort it to the extent suggested. Dr. W. D. Miller's last research showed most conclusively that much harm could be done by improper use of the tooth-brush and the dentifrice; but he was too wise to advocate the abolishing of these most important and useful prophylactic agents. Where one person abuses these toilet necessities dozens lose their teeth from neglect to use them. Agitation is good however.

